# Are there any differences between features of proteins expressed in malignant and benign breast cancers?

**Published:** 2010

**Authors:** Mansour Ebrahimi, Esmaeil Ebrahimie, Narges Shamabadi, Mahdi Ebrahimi

**Affiliations:** a Bioinformatics Research Group, Green Research Center, Qom University, Qom, Iran; b Department of Crop Production and Plant Breeding, College of Agriculture, Shiraz University, Shiraz, Iran; c Member of Young Researcher Club, Islamic Azad University of Qom, Qom, Iran; d Department of Informatics, Saarland University, Saarbrucken, Germany

**Keywords:** Computational Biology, Decision Support Techniques, Breast Neoplasms

## Abstract

**BACKGROUND:**

The most common cancer among women is breast cancer and it has been blamed as the second leading cause of cancer death in women; so far many approaches have been used to analyze and detect benign and malignant forms of cancer and understanding the features involved in proteins expressed by various types of breast cancers is crucial.

**METHODS:**

Herein features of proteins expressed in malignant, benign and both cancers were compared using different screening techniques, clustering methods, decision tree models and generalized rule induction (GRI) algorithms to look for patterns of similarity in two benign and malignant breast cancer groups.

**RESULTS:**

The findings showed that the N-terminal amino acid was Met and 57 out of 838 proteins’ features ranked as important (p > 0.05). The depth of the trees induced by tree induction models varied from 5 (in the Quest model) to 2 (in the C5.0 model) branches. The best performance evaluation found when C&RT model applied and the worst evaluation found when CHAID model applied. No significant difference in the percentage of correctness, performance evaluation, and mean correctness in tree induction algorithms was found when feature selection applied on datasets, but the number of peer groups reduced significantly (p < 0.05) when feature selection model applied.

**CONCLUSIONS:**

The frequency of Ile-Ile was the most important protein attributes in all tree and rule induction models. The importance of sequence-based classification and the frequency of Ile-Ile in prediction of malignant and benign breast cancer have been discussed here.

The most common cancer among women is breast cancer, excluding non-melanoma skin cancers and it is the second leading cause of cancer death in women (exceeded only by lung cancer); although recent studies confirmed death rates from breast cancer declined significantly during last decade.^1^ These declines may be due to earlier detection or better treatment. If the breast cancer is diagnosed early enough, the cure rate is very high and more than 97% of women can survive at least for 5 years.^2^ Generally the cancer has been categorized as non-invasive or benign, where the cancer cells are confined to the origin place, do not threaten life and do not spread outside of the breast; and invasive or malignant, where the cancer cells have broken through the duct into the surrounding fatty and connective tissues; this type may lead to death if not detected and cured.^3^

Although various techniques have been used to distinguish between benign and malignant breast cancers in recent years, use of computer based technologies such as bioinformatics models have attracted huge attentions.^4^–^7^ The Support Vector Machine (SVM) classification algorithm shown to be a useful tool to diagnose breast cancer.^8^,^9^ Bioinformatics tools such as feature selection with extensive RNA-pathway analysis on mass spectrometric of metabolites used to identify the important features related to breast cancer pathogenesis.^8^ In another attempt to introduce a predictive system for non-invasive breast cancer, a combination of another bioinformatics tools (SVM-based feature selection) and mass spectrometric analysis was employed.^10^ Neural network propagation algorithm with SVMs and other baseline methods were used to identify several markers with clinical or biological relevance with the breast cancers.^11^

Prediction tasks are attempts to accurately forecast the outcome of a specific situation by using input data obtained from a concrete set of variables that potentially describe the situation.^12^,^13^ Nowadays, neural networks, as artificial intelligence, have found application in a wide range of problems^14^ and in many cases resulted as superior to standard statistical models.^15^ The predictive reliability of an artificial neural networks model in medical diagnosis has been confirmed so far.^16^ Modeling systems have been used for better prediction of breast and lung carcinoma post-surgery survival using neural networks as suitable tool.^17^,^18^

When data analysis involve hundreds, or even thousands of variables, data mining tools are being used as one of the most probable candidates.^19^ It is anticipated that applying a neural network or a decision tree to a set of variables of this quantity may require more time than practice.^20^ There are many attributes determine the different characteristics of a protein molecule. As a result, the majority of time and effort of artificial modeling algorithms spent in the model-building process involves determining which variables should be included in the model. Attribute weighting or feature selection helps the model to reduce the size of variable set, extracting a more manageable set of attributes for rule or tree induction or getting out meaningful models.^21^ The value of a discrete dependent variable with a finite set from the values of a set of independent variables is predicted by induction tree algorithms.^22^ The tree is constructed by looking for regularities in data, determining the features to add at the next level of the tree using an entropy calculation, and then choosing the feature that minimizes the entropy impurity.^23^ There are many well-known decision tree algorithms available. To better understand the features that contribute to the type of proteins expressed in breast cancer (benign or malignant) and to find a suitable tool to classify the types of cancer according to proteins’ attributes, various clustering, screening, and decision tree models were employed in this study.

## Methods

From the UniProt Knowledgebase (Swiss-Prot and TrEMBL) database, sequences from 15 proteins expressed during two distinctive forms of breast cancers (10 benign and 5 malignant) and one common group (with 6 proteins in both benign and malignant groups) were retrieved. The proteins were categorized into B (benign), M (malignant) and C (control) groups. Eight hundred and seventy nine protein attributes or features such as length, weight, isoelectric point, aliphatic index, the count and the frequency of each amino acid and the count and the frequency of dipeptides from all of those proteins were calculated. All attributes were classified as continuous variables, except for the N-terminal amino acid, which was classified as categorical. A dataset of these protein features was imported into Clementine software (Clementine_NLV-11.1.0.95; Integral Solution, Ltd.), and type of cancer variable (B, M and C) was set as the output variable and the other variables were set as input variables.

Different tree induction algorithms were applied to the datasets to find the most important attributes and trace the most probable patterns expressed during two forms of cancers. These algorithms allowed the development of classification systems that automatically included in their rules only the attributes that really matter in making a decision. Attributes that did not contribute to the accuracy of the tree were ignored. This process yielded very useful information about the data and could be used to reduce the data to relevant fields only before training another learning technique, such as a neural network. Various algorithms are available for performing classification and segmentation analysis, and herein different decision tree and cluster analysis models were used. To investigate the effects of the attribute weighting algorithm on other models behavior, all models were run both with and without feature selection criteria.

Two screening models were used:

### 

#### a) Anomaly Detection Model:

By examining large numbers of attributes, this model was used to identify outliers or unusual cases in the data.

#### b) Attribute Weighting Algorithm:

This model identifies the features that have a strong correlation with the type of cancers and labels the attributes as important, marginal, and unimportant, with values more than 0.95, between 0.95 and 0.90, and less than 0.90, respectively.

Two clustering models applied:

#### a) K-Means:

This model clusters data into distinct groups when clustering groups are unknown. Records are grouped so that those within a group or cluster tend to be similar to each other, whereas records in different groups are dissimilar.

#### b) Two-Step Cluster:

In two-step cluster, the first step scans the data and compresses them into a manageable set of subclusters and in the second step a hierarchical clustering method applies to merge subclusters into larger clusters.

Five different tree induction models applied:

#### a) Classification and Regression Tree (C&RT):

This algorithm uses recursive partitioning to split the training records into segments by minimizing the impurity at each step.

#### b) CHAID:

Decision trees generated by using chi-square statistics to identify optimal splits.

#### c) Exhaustive CHAID:

A modification of CHAID with examining all possible splits.

#### d) QUEST:

A binary classification method generates and reduces the processing time.

#### e) C5.0:

A tree or a rule set induces by splitting the sample based on the field that provides the maximum information gain at each level.

Generalized rule induction (GRI) model or association model discovers association rules in the data by extracting a set of rules from the data using an index that takes both the generality (support) and accuracy (confidence) of rules into account.

## Results

The average length, weight, isoelectric point, and aliphatic indices of proteins studied here were 794.524 ± 749.528, 108.873 ± 112.782, 6.625 ± 1.238, and 84.914 ± 10.832 (mean ± SD), respectively. The average counts of sulfur, carbon, nitrogen, oxygen, and hydrogen were 33.810 ± 25.390, 4022.333 ± 3826.498, 1105.952 ± 1076.559, 1207.905 ± 1177.811, and 6373.667 ± 6128.062, respectively, and the average counts of hydrophobic, hydrophilic, and other residues were 359.857 ± 313.313, 201.952 ± 208.667, and 1160.000 ± 232.714, respectively. The frequencies of hydrogen, carbon, oxygen, nitrogen, and sulfur in all enzymes were 0.490 ± 0.009, 0.314 ± 0.054, 0.099 ± 0.006, 0.086 ± 0.004 and 0.002 ± 0.001, respectively, and the frequencies of hydrophobic, hydrophilic, other (amphoteric) residues, and negatively, positively, and other charged residues were 0.454 ± 0.059, 0.243 ± 0.037, 0.302 ± 0.081, 112.429 ± 116.015, 101.095 ± 99.609 and 581.000 ± 538.844, respectively. The mean count of amino acids ranged from a minimum of 11.095 ± 15.251 for Try to a maximum of 85.095 ± 92.529 for Leu and the same order found for amino acids frequencies (from 0.012 ± 0.007 for Try to 0.103 ± 0.023 for Leu). The N-terminal amino acid was Met among all proteins studied in this paper. The average non-reduced Cys extinction coefficient at 280 nm was 92462.381 ± 104156.36, non-reduced Cys absorption was 3380.877 ± 15488.905, the reduced Cys extinction coefficient was 88248.143 ± 105517.497, and the reduced Cys absorption was 0.915 ± 0.385.

### 

#### Screening Models

Two peer groups with an anomaly index cutoff of 1.352 were generated. No anomalous record found in the first peer group of 5 records, while 1 anomaly record found in the second peer group of 16 records. Two peer groups with an anomaly index cutoff of 1.53 and just 1 anomalous record in the second peer group created when feature selection algorithm applied on dataset.

Fifty seven out of 838 attributes had p value higher than 0.95 in classification of cancer proteins (Table 1), and 84 attributes with weight between 0.90 and 0.95 marked as marginal when feature selection model applied.

**Table 1:** Results of feature selection on important (and one marginal) features contributing to the two types of breast cancers proteins. Higher values indicate that protein feature is more important.

No	Protein feature	Value	Rank	No	Protein feature	Value	Rank
1	Count of Leu-Ile	0.998	Important	31	Count of Thr-Ile	0.972	Important
2	Count of Met-Ser	0.997	Important	32	Freq of Asp-Asp	0.972	Important
3	Freq of Asn-Ile	0.996	Important	33	Freq of Asn-Gln	0.972	Important
4	Count of Ile-Ile	0.995	Important	34	Count of Thr-Tyr	0.972	Important
5	Count of Ile-Cys	0.995	Important	35	Count of Leu-Cys	0.971	Important
6	Freq of Met-Ser	0.995	Important	36	Freq of Cys-Tyr	0.971	Important
7	Count of Ser-Phe	0.993	Important	37	Count of Asp-Tyr	0.971	Important
8	Count of Phe-Leu	0.992	Important	38	Count of Ile	0.968	Important
9	Freq of Gln-Val	0.991	Important	39	Count of Phe-Lys	0.967	Important
10	Count of Gly-Phe	0.99	Important	40	Count of Cys-Val	0.966	Important
11	Count of Gly-Val	0.989	Important	41	Count of Ser-Val	0.964	Important
12	Freq of Gly-Phe	0.988	Important	42	Count of Phe-Phe	0.963	Important
13	Count of Ala-Ile	0.987	Important	43	Freq of Glu-Trp	0.963	Important
14	Count of Tyr-Cys	0.986	Important	44	Count of Phe-Ile	0.962	Important
15	Count of Ile-Pro	0.986	Important	45	Freq of Phe-Met	0.962	Important
16	Freq of Asp-Leu	0.985	Important	46	Count of Gln-Val	0.961	Important
17	Count of Val-Ala	0.985	Important	47	Count of Asn-Arg	0.96	Important
18	Freq of Ile	0.984	Important	48	Count of Tyr-Trp	0.96	Important
19	Count of Phe (F)	0.981	Important	49	Count of Cys	0.959	Important
20	Count of Tyr-Pro	0.981	Important	50	Freq of Ala-Arg	0.958	Important
21	Freq of Asp-Ala (1)	0.98	Important	51	Count of Glu-Asn	0.956	Important
22	Count of Ala-Gly	0.979	Important	52	Freq of Ala-Ala	0.953	Important
23	Count of Val-Tyr	0.979	Important	53	Count of Ile-Asn	0.952	Important
24	Count of Lys-Phe	0.978	Important	54	Count of Asp-Arg	0.952	Important
25	Freq of His-Met	0.977	Important	55	Freq of Gln-Phe	0.951	Important
26	Count of Ile-Phe	0.976	Important	56	Count of Gly-Ile	0.951	Important
27	Freq of Ala-Leu	0.974	Important	57	Count of Asp-Lys	0.951	Important
28	Freq of Lys-His	0.973	Important	58	Count of Trp-Tyr	0.948	Important
29	Count of Phe-Asp	0.973	Important	59	Freq of Asn-Phe	0.947	Marginal
30	Freq of Asp(D)	0.973	Important	60	Count of Tyrosine (Y)	0.947	Marginal

#### Clustering Models

Six records (more than 28%) put into the first and the fourth clusters and 1, 5, and 3 records were put into the second, third, and fifth clusters, respectively when K-Means algorithm applied on the dataset and five clusters with 8, 1, 8, 3, and 1 records in each cluster, respectively, generated when feature selection filtering applied on dataset.

Two clusters with 1 and 20 records in each group, respectively, generated when Two-Step clustering applied on dataset without feature selection and again two clusters (with 5 and 16 records in each cluster) created when feature selection algorithm applied.

#### Decision Tree Models

**Figure 1:**
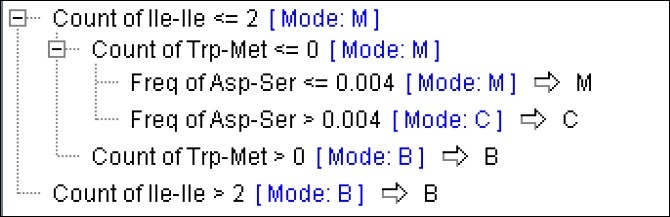
A decision tree generated by the CHAID modeling method without feature selection filtering showing protein features used to build the decision tree. M = Malignant cancer; B = Benign; C = Common proteins

A tree with a depth of 2 and cross-validation of 45.0 ± 9.0 induced in C5.0 model and the most important attribute employed to build the tree was the count of Ile-Ile. If the value of this feature was equal to or less than 2, the proteins fell into the malignant (M) category; otherwise they were put into the benign (B) category. In the M subgroup, the frequency of Arg-Cys was used to create the next tree branches, with value equal to 0 as M mode and more than 0 as common (C) mode. When 10-fold cross-validation was applied to the same dataset, again a tree with a depth of 2 and cross-validation of 56.7 ± 11.7 was created. The same protein features and values were used to create tree branches. When the same models were applied to datasets using feature selection filtering, a tree with a depth of 3 and cross-validation of 58.3 ± 10.3 and 58.3 ± 7.1 were generated for C5.0 and C5.0 with 10-fold cross-validation, respectively. Again the count of Ile-Ile (with value of 2) was used to create the first tree branches while count of Ile-Cys was the feature used for second subgroups classification with values equal to or greater than 0 (Figure 1).

A tree with a depth of 3 induced when C& RT model applied and the most important attribute to build the tree was the count of Ile-Ile (value ≥ 2.500 for M and > 2.500 for B groups). The frequency Asp-Ser was used to create the second level for M subgroups (with value of 0.004). The same results were obtained when feature selection was used.

A tree with a depth of 5 generated when Quest model applied and the frequency of His-Met (with a value of 0) was the most important feature to create the first tree branches. In the M subgroup, count of Cys-Gln (0), the count of Ile-Met (value 0) and the count of Gln-His (value 0.691) were the most important features in creating the subsequent branches of M group decision tree. Nearly the same results were obtained when feature selection filtering was applied.

**Figure 2:**
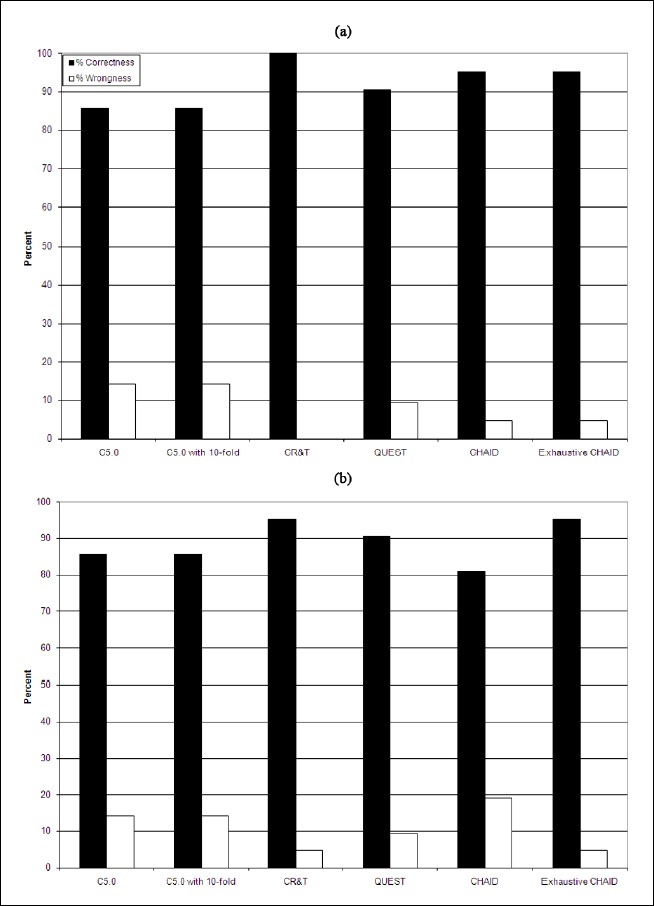
Percentage of correctness and wrongness in various decision tree models, in datasets without feature selection (a) and with feature selection (b); showing C5.0 model had the best performance, followed by CR&T, CHAID, and Quest models

In CHAID model with and without feature selection, a tree with a depth of 3 induced and again the same protein feature (count of Ile-Ile) with the same values as C5.0 model used to create the tree. The same trees with the same features and values were generated when exhaustive CHAID models were applied on data-sets with and without feature selection. The best percentage of correctness, performance evaluation, and mean correctness in the tree induction models belonged to C5.0 model, followed by the CR&T, CHAID, and finally the Quest models (Table 2 and Figure 2).

**Table 2:** The association rules found in the data by the generalized rule induction (GRI) method, showing 100 most important rules created by GRI algorithm in classifying benign (B), malignant (M) and common (C) proteins expressed in breast cancers.

Antecedent	Support (%)
Count of Ile-Ile > 2.500	42.86
Count of Ile > 27.000 and Freq of Ala-Ala < 0.004	42.86
Count of Phe-Lys > 0.500 and Count of Ile > 28.500	38.1
Count of Ala-Gly > 0.500 and Count of Ile-Cys > 0.500	38.1
Count of Ile > 27.000 and Count of Tyr-Pro > 0.500	38.1
Freq of Met-Ser < 0.000 and Freq of Gln-Val < 0.002 and Freq of Asp < 0.078	23.81
Count of Asp-Arg > 1.500	33.33
Count of Asp-Lys > 2.500 and Freq of Ala-Ala < 0.004	33.33
Count of Cys-Val > 1.500 and Count of Ile > 30.000	33.33
Count of Ala-Gly > 0.500 and Count of Asp-Arg > 1.500	33.33
Count of Ala-Gly > 0.500 and Freq of Asp < 0.059 and Freq of Ala-Ala < 0.004	33.33
Count of Ala-Gly > 0.500 and Count of Ile > 28.500 and Freq of Ala-Leu < 0.008	33.33
Count of Ile > 27.000 and Count of Tyr-Cys < 1.500 and Freq of Ala-Ala < 0.004	33.33
Count of Ile > 27.000 and Count of Val-Ala < 7.000 and Freq of Ala-Ala < 0.004	33.33
Count of Ile > 27.000 and Count of Ser-Val < 5.500 and Freq of Ala-Ala < 0.004	33.33
Count of Ile > 27.000 and Count of Gln-Val < 6.000 and Freq of Ala-Ala < 0.004	33.33
Count of Ile > 27.000 and Count of Asn-Arg < 4.500 and Freq of Ala-Ala < 0.004	33.33
Count of Ile > 27.000 and Count of Met-Ser < 2.500 and Freq of Ala-Ala < 0.004	33.33
Count of Ile > 27.000 and Count of Lys-Phe < 4.500 and Freq of Ala-Ala < 0.004	33.33
Count of Ile > 27.000 and Count of Ile-Pro < 2.500 and Freq of Ala-Ala < 0.004	33.33
Count of Ile > 27.000 and Count of Ile-Ile < 5.500 and Freq of Ala-Ala < 0.004	33.33
Count of Ile > 27.000 and Count of Gly-Ile < 5.500 and Freq of Ala-Ala < 0.004	33.33
Count of Ile > 27.000 and Count of Gly-Phe < 3.500 and Freq of Asp-Leu < 0.006	33.33
Count of Ile > 27.000 and Count of Phe-Lys < 4.000 and Freq of Ala-Ala < 0.004	33.33
Count of Ile > 27.000 and Count of Phe-Phe > 0.500 and Freq of Ala-Leu < 0.008	33.33
Count of Ile > 27.000 and Count of Glu-Asn < 3.500 and Freq of Ala-Ala < 0.004	33.33
Count of Ile > 27.000 and Count of Asp-Arg < 4.500 and Freq of Ala-Ala < 0.004	33.33
Count of Ile > 27.000 and Count of Asp-Lys < 6.500 and Freq of Ala-Ala < 0.004	33.33
Count of Ile > 27.000 and Count of Ala-Ile < 7.000 and Freq of Ala-Ala < 0.004	33.33
Count of Ile > 27.000 and Count of Ala-Gly < 3.500 and Freq of Ala-Ala < 0.004	33.33
Count of Ile > 27.000 and Freq of Asp < 0.056 and Freq of Ala-Ala < 0.004	33.33
Count of Ile > 27.000 and Count of Phe < 64.500 and Freq of Ala-Ala < 0.004	33.33
Freq of Asn-Ile < 0.000 and Freq of Ala-Ala < 0.008 and Count of Asp-Lys > 1.500	14.29
Freq of Asn-Ile < 0.000 and Count of Asp-Lys > 1.500 and Freq of Ala-Ala < 0.008	14.29
Freq of Asn-Ile < 0.000 and Freq of Ile > 0.040 and Freq of Ala-Leu < 0.008	14.29
Freq of Asn-Ile < 0.000 and Count of Ile > 11.500 and Freq of Ala-Leu < 0.008	14.29
Freq of Gly-Phe < 0.000 and Freq of Ala-Ala < 0.008 and Count of Ile > 11.500	14.29
Freq of Gly-Phe < 0.000 and Count of Asp-Lys > 1.500 and Freq of Ala-Ala < 0.008	14.29
Count of Ala-Ile < 1.500 and Freq of Ala-Ala < 0.008 and Count of Ile > 11.500	14.29
Count of Ala-Ile < 1.500 and Count of Ile-Ile > 0.500 and Freq of Ala-Ala < 0.008	14.29
Count of Ala-Ile < 1.500 and Count of Asp-Lys > 1.500 and Freq of Ala-Ala < 0.008	14.29
Count of Ile < 25.500 and Freq of Ala-Ala < 0.008 and Count of Ile > 11.500	14.29
Count of Ile < 25.500 and Count of Ile-Ile > 0.500 and Freq of Ala-Ala < 0.008	14.29
Count of Ile < 25.500 and Count of Asp-Lys > 1.500 and Freq of Ala-Ala < 0.008	14.29
Count of Asp-Lys > 2.500 and Count of Asp-Tyr > 1.500	28.57
Count of Asp-Lys > 2.500 and Freq of Ile < 0.066 and Freq of Ala-Ala < 0.004	28.57
Count of Ala-Ile > 2.500 and Freq of Asp-Leu < 0.006	28.57
Count of Ala-Gly > 0.500 and Freq of Ile > 0.059	28.57
Count of Ala-Gly > 0.500 and Freq of Asp < 0.059 and Freq of Asp > 0.044	28.57
Count of Ala-Gly > 0.500 and Count of Cys > 14.000 and Freq of Ala-Leu < 0.006	28.57
Freq of Ile > 0.059 and Count of Ala-Gly > 0.500	28.57

Count of Ile > 27.000 and Count of Gly-Ile < 5.500 and Count of Tyr-Pro > 0.500	28.57

Count of Ile > 27.000 and Count of Gly-Phe < 3.500 and Count of Phe-Lys > 1.500	28.57
Count of Ile > 27.000 and Count of Phe-Lys < 4.000 and Count of Cys-Val > 1.500	28.57
Count of Ile > 27.000 and Count of Phe-Asp < 4.000 and Freq of Ala-Ala < 0.004	28.57
Count of Ile > 27.000 and Count of Glu-Asn < 3.500 and Count of Cys-Val > 1.500	28.57
Count of Ile > 27.000 and Count of Asp-Arg < 4.500 and Count of Glu-Asn > 1.500	28.57
Count of Ile > 27.000 and Count of Asp-Lys < 6.500 and Count of Cys-Val > 1.500	28.57
Count of Ile > 27.000 and Count of Cys-Val < 2.500 and Freq of Ala-Ala < 0.004	28.57
Count of Ile > 27.000 and Count of Ala-Ile < 7.000 and Count of Cys-Val > 1.500	28.57
Count of Ile > 27.000 and Freq of Asp < 0.056 and Count of Asn-Arg > 1.500	28.57
Count of Ile > 27.000 and Count of Phe < 64.500 and Count of Cys-Val > 1.500	28.57
Count of Ile > 27.000 and Count of Cys < 28.500 and Freq of Ala-Ala < 0.004	28.57
Freq of Met-Ser < 0.000 and Freq of Lys-His < 0.002 and Count of Thr-Ile > 0.500	19.05
Freq of Met-Ser < 0.000 and Freq of Phe-Met < 0.002 and Count of Thr-Ile > 0.500	19.05
Freq of Met-Ser < 0.000 and Freq of Asp-Leu < 0.013 and Count of Asn-Arg < 0.500	19.05
Freq of Met-Ser < 0.000 and Freq of Asp-Ala (1) < 0.014 and Count of Asn-Arg < 0.500	19.05
Freq of Met-Ser < 0.000 and Freq of Asp-Asp < 0.004 and Count of Thr-Ile > 0.500	19.05
Freq of Met-Ser < 0.000 and Count of Thr-Ile > 0.500 and Count of Asn-Arg < 0.500	19.05
Freq of Met-Ser < 0.000 and Count of Asn-Arg < 0.500 and Count of Cys > 1.500	19.05
Freq of Met-Ser < 0.000 and Count of Leu-Ile > 0.500 and Count of Asn-Arg < 0.500	19.05
Freq of Met-Ser < 0.000 and Freq of Asp < 0.070 and Count of Asn-Arg < 0.500	19.05
Freq of Cys-Tyr < 0.000 and Freq of Lys-His < 0.002 and Count of Thr-Ile > 0.500	19.05
Freq of Cys-Tyr < 0.000 and Freq of Phe-Met < 0.002 and Count of Cys > 1.500	19.05
Freq of Cys-Tyr < 0.000 and Freq of Asp-Leu < 0.013 and Count of Asn-Arg < 0.500	19.05
Freq of Cys-Tyr < 0.000 and Freq of Asp-Ala (1) < 0.014 and Count of Asn-Arg < 0.500	19.05
Freq of Cys-Tyr < 0.000 and Freq of Asp-Asp < 0.004 and Count of Thr-Ile > 0.500	19.05
Freq of Cys-Tyr < 0.000 and Count of Thr-Ile > 0.500 and Count of Asn-Arg < 0.500	19.05
Freq of Cys-Tyr < 0.000 and Count of Asn-Arg < 0.500 and Count of Cys > 1.500	19.05
Freq of Cys-Tyr < 0.000 and Count of Leu-Ile > 0.500 and Count of Asp-Arg < 0.500	19.05
Freq of Cys-Tyr < 0.000 and Freq of Asp < 0.070 and Count of Asn-Arg < 0.500	19.05
Count of Met-Ser < 0.500 and Freq of Ala-Leu > 0.008	19.05
Count of Met-Ser < 0.500 and Count of Asn-Arg < 0.500 and Freq of Ala-Leu > 0.008	19.05
Count of Met-Ser < 0.500 and Freq of Asp < 0.070 and Freq of Ala-Leu > 0.008	19.05
Count of Asp-Lys > 2.500 and Count of Asp-Arg < 4.500 and Freq of Ile > 0.048	23.81
Count of Asp-Lys > 2.500 and Count of Ala-Ile < 7.000 and Count of Leu-Ile > 3.500	23.81
Count of Asp-Lys > 2.500 and Count of Ala-Gly < 3.500 and Freq of Ala-Ala < 0.004	23.81
Count of Asp-Lys > 2.500 and Freq of Ile < 0.066 and Count of Asp-Tyr > 1.500	23.81
Count of Asp-Lys > 2.500 and Freq of Asp < 0.056 and Freq of Asp > 0.044	23.81
Count of Asp-Lys > 2.500 and Count of Ile < 85.500 and Freq of Ile > 0.048	23.81
Count of Asp-Lys > 2.500 and Count of Phe < 64.500 and Count of Leu-Ile > 3.500	23.81
Count of Asp-Lys > 2.500 and Count of Cys > 14.000 and Freq of Ala-Leu < 0.006	23.81
Count of Cys-Val > 1.500 and Count of Cys > 19.000	23.81
Count of Ala-Ile > 2.500 and Count of Val-Tyr > 2.000	23.81
Count of Ala-Ile > 2.500 and Count of Val-Ala < 9.500 and Freq of Asp-Leu < 0.006	23.81
Count of Ala-Ile > 2.500 and Count of Thr-Tyr < 2.500 and Freq of Asp-Leu < 0.006	23.81
Count of Ala-Ile > 2.500 and Count of Thr-Ile < 6.500 and Freq of Asp-Leu < 0.006	23.81
Count of Ala-Ile > 2.500 and Count of Ser-Val < 8.000 and Freq of Asp-Leu < 0.006	23.81
Count of Ala-Ile > 2.500 and Count of Ser-Phe < 5.500 and Freq of Asp-Leu < 0.006	23.81

#### Association Model

One hundred rules with 21 valid transactions and minimum and maximum support of 47.62% and 83.23% and maximum confidence of 100 % generated when GRI model applied. When feature selection was used, minimum support, maximum support, maximum confidence, and minimum confidence changed to 14.29%, 42.86%, 100%, and 87.5%, respectively.

When the most accurate model (C&RT) was run on another dataset of 30 proteins from other cancers, the accuracy of the model in predicting the right group was 95.24%, while its wrongness was just 7.76% showing very suitable performance of this model in prediction.

## Discussion

Nowadays, incredible amount of data produced each year because cancer research is a worldwide enterprise and the application of computational tools in cancer research has become an important and rapidly developing field. Bioinformatics as an emerging tool has developed primary to address the analysis of huge data generated from genomics and proteomics; however large datasets are also produced in cell biology, physiology, pathology, therapeutics, clinical trials and epidemiology.

To utilize and improve the extraction of valuable results generated by researchers on patients’ diagnosis and treatment, collaboration between various sections of sciences such as software engineering, data mining knowledge and clinical studies seems essential.^24^–^26^

Early diagnosis of breast cancer is much more significant than any treatment, therefore, more attention should be paid to the early diagnosis of breast cancer.^27^ Self-examination, clinical examination, physical examination and mammography are main diagnostic tools but these classical methods are useful when tumours are large or palpable and mammography, as an efficient tool, is mainly suitable in western countries. Use of serum markers such as CA15.3, CA27.29 and CEA, without enough sensitivity and specificity has not been accepted in clinical diagnostics; especially in the early stages of breast cancers. Although Food and Drug Administration (FDA) of the United States recommended some markers only for monitoring therapy or recurrence of advanced breast cancer; it is been highly recommended to find new diagnostic tools; and some researchers have proposed proteomics and bioinformatics approaches as emerging tools for breast cancer detection.^26^,^28^ These tools in conjunction with bioinformatics applications could greatly facilitate the discovery of new and better biomarkers.^25^

Various modelling tools (Screening Models, Clustering Models, Decision Tree Models and Association Model) applied on more than 800 protein attributes expressed in benign, malignant and both types of breast cancers simultaneously to find different protein features in each class of breast cancers. The screening, clustering, and decision tree models applied on datasets with and without feature selection filtering.

Although 85 attributes (with value greater than 0.95) were marked as “important”, more than 95% of them were the frequencies or the counts of dipeptides. The number of peer groups with anomalies did not change when feature selection algorithm were applied, showing the neutral effects of attribute weighting on removing outliers in this case; although in another study we showed feature selection significantly improves the performances of the modelling in classifying mesostable and thermostable proteins.^29^ In K-Means modelling, the number of clusters did not show any differences when models run on dataset with and without feature selection, although the number of records in the clusters changed.

The depth of trees varied from 5 (in the Quest model) to 2 (in the C5.0 with and without 10-fold cross validation models) branches when tree induction models applied. The best performance evaluation belonged to C&RT model and the worst to C5.0 and C5.0 models with 10-fold cross validation. The percentage of correctness, performance evaluation, and mean correctness of tree induction models applied here showed no significant differences (p > 0.95) with and without feature selection filtering on datasets, but when feature selection datasets used the percentage of correctness of CAHID model decreased.

In all tree induction models, the count of Ile-Ile chose as the most important attribute and also in all GRI association rules (100 rules) the count of this feature was used as an antecedent to support the rules. A consistent difference exists in the pattern of synonymous codon usage between benign and malignant protein cancers,^30^,^31^ and there is strong evidence that this difference is the result of selections linked to malignancy coming out from amino acid sequences.^32^,^33^ In addition, malignant proteins can be distinguished based on the amino acid composition of their proteomes, and several authors have tried to relate these differences to structural differences.^34^–^38^

The importance of sequence-based classification in detection of various proteins expressed in breast cancer and the importance of Ile-Ile dipeptide in clustering of proteins, for the first time, reported in this paper. As Ile is a non-polar and hydrophobic amino acid, when it forms a dipeptide bond, it clearly can change the confirmation of proteins so that it has been used as the most important feature in all decision tree models applied in this paper.

The performance of different bioinformatics tools (such as screening, clustering, and decision tree algorithms) for discriminating between proteins expressed in malignant and benign types of breast cancer examined here. The results confirmed that amino acid composition can be used to discriminate between proteins groups expressed in two forms of breast cancer. The results also confirmed that most of algorithms employed here can be used to discriminate between proteins expressed in two main forms of breast cancers with an accuracy of 86-100%. No significant difference was found in performance of different models used in this paper. Interestingly, the CHAID and exhaustive CHAID methods showed lower performance in comparison with other decision tree models as we anticipated to be more accurate, because they use the most sophisticated neural network architecture and trim it down to desired level, so the number of hidden layers and the number of neurons in layers 1 and 2 are usually higher than other decision tree models. When feature selection applied no significant differences (p > 0.05) noticed between analyses. The best performance and results were obtained with C&RT algorithms. Thus, it is suggested that this decision tree model can be used as an effective tool to discriminate malignant and benign proteins of breast cancer.

## Conclusions

In this study a new approach has been employed for the first time to look at the protein attributes’ variations in malignant and benign breast cancers. The frequency of Ile-Ile was the most important protein attributes in all tree and rule induction models.
